# Meeting the Challenges of High-Dimensional Single-Cell Data Analysis in Immunology

**DOI:** 10.3389/fimmu.2019.01515

**Published:** 2019-07-03

**Authors:** Subarna Palit, Christoph Heuser, Gustavo P. de Almeida, Fabian J. Theis, Christina E. Zielinski

**Affiliations:** ^1^TranslaTUM, Technical University of Munich, Munich, Germany; ^2^Institute of Virology, Technical University of Munich, Munich, Germany; ^3^Partner Site Munich, German Center for Infection Research, Munich, Germany; ^4^Institute of Computational Biology, Helmholtz Zentrum München, Neuherberg, Germany; ^5^Department of Mathematics, Technical University of Munich, Munich, Germany

**Keywords:** high-dimensional data analysis, CyTOF, single-cell profiling, single-cell genomics, visualization, trajectory inference, systems immunology

## Abstract

Recent advances in cytometry have radically altered the fate of single-cell proteomics by allowing a more accurate understanding of complex biological systems. Mass cytometry (CyTOF) provides simultaneous single-cell measurements that are crucial to understand cellular heterogeneity and identify novel cellular subsets. High-dimensional CyTOF data were traditionally analyzed by gating on bivariate dot plots, which are not only laborious given the quadratic increase of complexity with dimension but are also biased through manual gating. This review aims to discuss the impact of new analysis techniques for in-depths insights into the dynamics of immune regulation obtained from static snapshot data and to provide tools to immunologists to address the high dimensionality of their single-cell data.

## The Challenge of Dimensionality in Manual Gating

Since the invention of the first fluorescence-based flow cytometer 50 years ago, immunologists have widely adopted the technology to get a comprehensive understanding of heterogeneity among immune cells, function, cellular differentiation, signaling pathways, and biomarker discovery (1). A variation of flow cytometry, known as cytometry by time-of-flight (CyTOF) or mass cytometry, was developed in 2009, which could query over 50 parameters per cell, in contrast to only limited parameters in conventional flow cytometry. CyTOF utilizes antibodies labeled with rare earth metal isotopes instead of fluorescent dyes and the resulting abundances are detected using a time-of-flight mass spectrometer (2, 3). The preferred high-throughput method for measuring cell surface or intracellular protein abundances depends on certain characteristics that distinguish one technology over the other (Table 1).

**Table 1:** Toward higher dimensions with CyTOF.

**Flow cytometry**	**Mass cytometry**
Spectral overlap between fluorophores necessitates data compensation and limits the number of measured markers (4)	CyTOF uses heavy metal isotopes; mitigates the issues associated with spectral overlap and auto-fluorescence
Can measure up to 20 parameters per cell with conventional flow cytometers	Queries over 50 markers per cell simultaneously
Cells can be further sorted for functional studies	Cells are destroyed during ionization
Highest throughput with tens of thousands of cells per second at relatively low operating costs	High dimension of parameter measurement at a lower throughput of hundreds of cells per second

Analysis of high-dimensional single-cell cytometry data relies on technological advancements and novel analytical methods that can efficiently incorporate the inherent multi-parametric characteristics of such data sets. The most straightforward and traditional, albeit labor-intensive, method for cytometry data analysis is by a process known as “gating,” which uses a series of 2D plots to identify regions of interest in a bivariate scatter plot of single cells (5). A series of gates drawn in sequence can reveal information about cellular hierarchy and identify subsets of interest from a population. Nevertheless, this approach has several drawbacks when compared to automated strategies (Table 2). Data analysis can be handled in one of several ways as new methods are continuously emerging and the shortcomings of manual gating can jeopardize the validity of an experimental finding. A number of automated gating strategies were developed for this purpose. They were designed for cell population identification reproducing expert manual gating and also for sample classification according to some external variables. Several of these methods performed well statistically when compared to manual gating and have been reviewed extensively as part of the first FlowCAP challenge (http://flowcap.flowsite.org/) (6).

**Table 2:** The challenge of dimensionality in manual gating.

**Manual gating**	**Automated gating**
Depends highly on the investigator's knowledge, adding a potential bias	Based on unsupervised clustering and is therefore unbiased
Not easily scalable and data interpretation gets confounded as dimensionality increases	Can efficiently visualize distribution of every possible marker with fewer plots
High-dimensional data visualization requires multiple biaxial plots, which increase quadratically with number of measured parameters (dimensionality explosion).	Minimizes loss of relevant information through rare event detection and instantaneous assessment of all markers included

Another rapidly evolving technology, which is taking center stage, is single-cell RNA sequencing (scRNA-seq). It can comprehensively profile the molecular information of individual cells and provides transcriptomics as an additional orthogonal modality, with almost full genomic coverage (7). Despite potential challenges, rapid computational advancements in single-cell analysis have significantly enabled systematic investigations into cellular heterogeneity, dynamics as well as regulatory mechanisms in an increasing number of tissues.

## Paving the Way for More Complex Analyses

**Figure 1:**
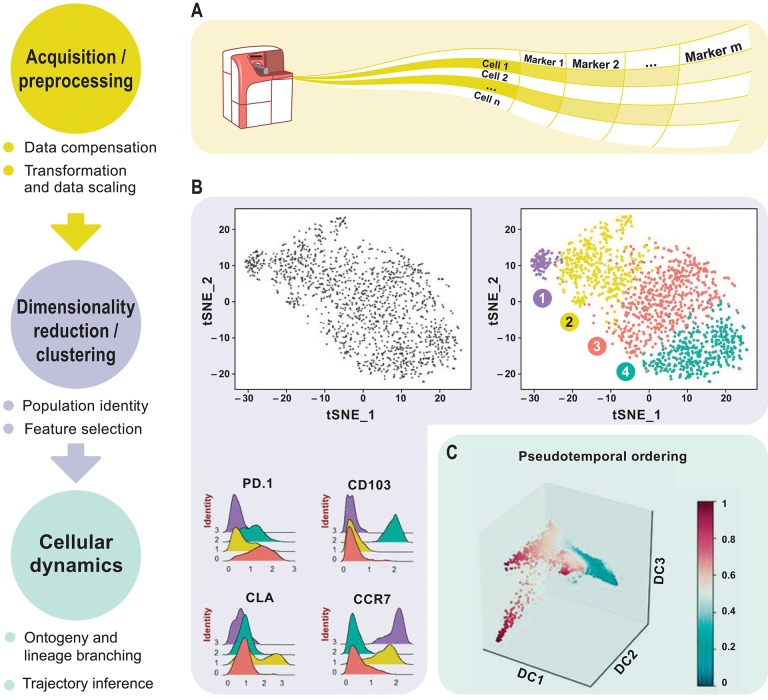
Overview of high-dimensional flow cytometry data analysis. **(A)** FCS files are read as the input data matrix, followed by data compensation (if necessary), transformation, concatenation (in case of multiple input files), and scaling. As an alternative to the standard log transformation method, logicle, and arcsinh transformation can be applied. Scaling ensures that every marker in the matrix gets equal importance in further downstream analyses. **(B)** For subsequent analyses, informative markers, such as lineage-specific markers, need to be selected. This can be accomplished via diagnostic plots for marker expression distribution across all events or with prior knowledge. Dimensionality reduction using PCA, t-SNE, diffusion maps etc. for visualization follows after feature selection. These plots show similarities between events, measured in an unsupervised manner, and provide information about differential expression between groups based on the intensity measurements. This reveals cellular heterogeneity. **(C)** Detection of developmental trajectories is another common application of these analytical frameworks and provides dynamic information on static snap shot data.

This review aims to discuss newly emerged data analysis tools for downsizing the above-mentioned drawbacks. Analyzing complex high-dimensional single-cell data comes with its own computational challenges and can be reduced to data pre-processing, normalization, dimensionality reduction, and clustering followed by cluster biomarker identification (Figures 1A–C). Many analysis tools that exist for scRNA-seq data can already be applied to cytometry studies with certain parameters optimized accordingly. To enable transition from experiment to data analysis, many algorithms have already been deployed in the form of interactive visual tools for bench scientists without a need of programming skills. This review delivers a guide for cytometry data analysis by discussing some of the available algorithms including, but not limited to, t-SNE (8), diffusion maps (9), SPADE (10), and FlowSOM (11). For the first time, we propose two single-cell trajectory inference algorithms, diffusion pseudo-time (DPT) (12) to infer pseudo-temporal ordering of cells and partition-based graph abstraction (PAGA) (13) for generating network topologies according to relative protein abundances from cytometry data (Table 3). Originally proposed for the analysis of scRNA-seq, we believe these algorithms hold great potential to uncover the immune system's cellular composition and differentiation trajectories in a heuristic manner given the growth in dimensions.

**Table 3:** Overview of some of the established single-cell analysis methods.

**Class**	**Methods**	**Description**
Linear dimensionality reduction	PCA	Cannot account for the smooth nature of single-cell data
Non-linear dimensionality reduction	t-SNE	More intuitive representation of high-dimensional data on a lower manifold
	UMAP	Scales better and improves global structure of the data compared to t-SNE(see Box 1)
	HSNE	Scales better than conventional t-SNE(see Box 1)
	Diffusion maps	Explores continuity through progression of cell differentiation
Clustering methods; single-cell resolution is lost	SPADE	Hierarchical branched tree representation (see Box 2)
	FlowSOM	Self-organizing maps trained to detect cell populations (see Box 3)
Trajectory inference and graph abstraction	Diffusion pseudotime (DPT)	Investigates continuous cellular differentiation trajectories from static snapshot single-cell data (see Box 4)
	Partition-based graph abstraction (PAGA)	Reconstitutes topological information from complex differentiation single-cell data in the form of cellular maps by applying strategies of clustering and trajectory inference

## Visualizing Cellular Heterogeneity by Dimensionality Reduction

Flow cytometry represents one of the most powerful and frequently used technologies in the immunologist's toolbox. Single-cell resolution has been a hallmark of immunological data acquisition and analysis for decades. One of the goals of performing differential analyses of cytometry data sets is cellular sub-type classification. Clustering is one of the most challenging steps as it forms a basis for all subsequent differential tests on marker expression for biomarker discovery and population abundance analysis. Identification of cell populations depends on the number of features measured and cytometry has been able to push the detection limit to over 50 parameters per cell. However, increased dimensionality makes it difficult to capture the underlying heterogeneity of the data. Dimensionality reduction methods maximize the variance in the data and reduce the number of variables by mapping it onto a lower-dimensional space. Principal component analysis (PCA), a linear dimensionality reduction method, represents the original data in 2 or 3 dimensions by using a linear combination of the original feature vectors and maps data points onto orthogonal dimensions, which explain the maximum variance. However, PCA fails to capture the non-linear nature of single-cell data, which is better visualized using non-linear dimensionality reduction techniques like t-SNE or uniform manifold approximation and projection (UMAP) (14, 15) (Box 1). t-SNE represents each cell in a lower dimensional manifold that is computed using the Barnes-Hut implementation of the t-stochastic neighbor embedding (t-SNE) algorithm (18). t-SNE is currently one of the most popular methods of representing single-cell data.

Box 1 **Non-linear dimensionality reduction methods scaling better than t-SNE**

A recently introduced analysis technique for high-dimensional cytometry data known as HSNE or Hierarchical Stochastic Neighbor Embedding transcends the scalability limit of conventional t-SNE (16). HSNE constructs a hierarchy of non-linear similarities between events that can be explored interactively up to single-cell details. The utility of this technique is to identify rare cell types that might otherwise be missed during down-sampling. HSNE has been implemented by van Unen et al. (16) as an integrated analysis tool in Cytosplore (17).

Another non-linear dimensionality reduction technique, which has garnered special attention is UMAP (14, 15). It is based on a novel manifold learning technique and can preserve local neighborhood relationships, while it excels at retaining the global structure. Neighboring cells in the low-dimensional representation are also closely related in the high-dimensional space, which is not necessarily the case for t-SNE. Also, it scales better than most t-SNE packages in embedding large high-dimensional datasets while providing good resolution of rare cell types, such as those in transition, which makes UMAP a viable choice as a general-purpose non-linear dimensionality reduction method.

Briefly, t-SNE computes a pairwise similarity matrix between all cells using a distance metric calculated from the feature vectors in high dimensions. Next, it initializes each cell to a random starting location in the 2 or 3 t-SNE dimensions and computes a second lower dimensional similarity matrix. The algorithm tries to iteratively minimize the difference between the lower and higher dimensional similarity matrices thereby updating the location of each cell in 2 or 3 dimensions. Thus, the number of iterations is an important parameter that needs to be sufficiently large in order to reach a stable configuration. The second critical parameter is perplexity which, in simplified terms, is a measure to weigh local similarities vs. global similarities in the generation of the low-dimensional representation of the high-dimensional space. While perplexity values between 5 and 50 have been suggested (17), adequacy needs to be tested for the respective data-set (19). t-SNE optimization follows a stochastic nature, therefore every compilation of the method leads to slightly different lower manifold projections. It is advisable to run the method multiple times in order to achieve a concise representation of the variability of the different results. Furthermore, cells that are alike in higher dimensions are usually clustered together in t-SNE space. However, the opposite is not always true and thus this warrants caution with the analysis of t-SNE plots (also see Box 1).

**Figure 2:**
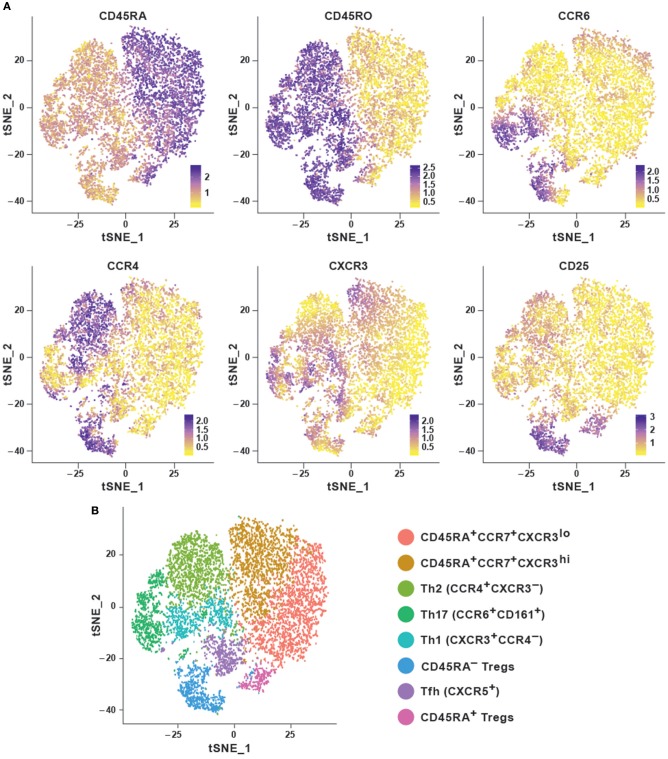
Dimensionality reduction using t-SNE shows cellular heterogeneity. **(A)** t-SNE plots for CD4^+^ PBMC (sample 1) from publicly available original CyTOF data (20) generated using the default parameters of the Rtsne package and colored by selected marker intensities (median expression) from high (purple) to low (yellow). **(B)** Identification of major immune subsets defined on the basis of lineage marker expression from **(A)**. Clusters were identified using the Seurat R package using the first eight principal components and a resolution of 0.6. Memory cells were then identified as CD45RA^−^CD45R0^+^ and further subset according to their chemokine receptor profile, naïve cells as CD45RA^+^CCR7^+^, and regulatory T cells as CD25^hi^CD127^lo^.

In order to explore the features of a t-SNE analysis, we applied the Rtsne package in R on an original CyTOF data-set (20). This data-set measures 40 surface markers in unstimulated cells (“Trafficking Panel”) and a combination of surface and intracellular targets following a brief *ex vivo* re-stimulation (“Trafficking and Function Panel”) in healthy human T cells from four lymphoid and four non-lymphoid tissues. Here, we used a pre-gated CD4^+^ T cell sample from peripheral blood mononuclear cells (PBMC) stained with the “Trafficking Panel” which includes markers for the identification of major T cell lineages and homing molecules. Our goal was to substantiate whether t-SNE was capable of recovering the major T-cell subtypes using a combination of surface markers and secreted cytokines. t-SNE cannot conveniently process very large data-sets and suffers from slow computation time. Since cytometry allows high-resolution dissection of cellular parameters, it usually measures a much larger number of cells, which can significantly increase computational complexities of the analyses. Down-sampling the data set usually overcomes such complexities. Density-dependent down-sampling detects regions of density within the data-set and then downsizes keeping the structure and distribution consistent. This is beneficial for rare cell populations to define their own clusters instead of being subsumed under the highly abundant cell types. We performed density-dependent down-sampling of the PBMC CyTOF dataset using the SPADE (10) package. The algorithm was run using lineage markers and default parameter settings. We observed that the surface markers could delineate most of the different subsets. We could define the different memory subsets within PBMC using marker profiles thereby providing information about the potential for cells to home and migrate (Figures 2A,B). Moreover, overlapping clusters suggest that CD4^+^ T cells are plastic and display the ability to differentiate from one to another subtype. t-SNE has been implemented in CRAN (http://www.r-project.org/) and it is also available as a plugin in FlowJo as well as in Cytobank at www.cytobank.org. Markers selected for t-SNE analyses may represent proteins that delineate classic cellular lineages and differentiation states. Different sequential t-SNE runs using identical numbers of iterations and perplexity result in slightly different results due to differing events sampling conditions. Despite of this, multiple runs on the same dataset will produce highly similar results with a preserved neighborhood structure.

Diffusion maps, similar to t-SNE, are mainly used for data visualization in a non-linear fashion and can be a classic tool to investigate continuity in single-cell data. They were introduced by Coifman and Lafon (21) and are constructed using eigenvalue decomposition of a random-walk based transition matrix, which was recently adapted in Haghverdi et al. to the single-cell setting (9). The method preserves the global relations between cells and has been able to successfully capture the developmental trajectories of differentiating cells, along with branching events, enabling it to capture both abundant as well as rare cell populations.

## Organizing Single cells as Clusters for Sub-type Classification

Many tools now exist that group cells into discrete sub-populations based on feature space such as SPADE, FlowSOM etc. and employ unsupervised techniques for visualization of high-dimensional cytometry data. SPADE or spanning-tree progression analysis of density-normalized events organizes cellular populations into hierarchies based on similar phenotypes (10). It provides an intuitive 2D depiction of multiple cell-types in a branched tree structure (Box 2).

Box 2 **Hierarchical tree representation of single-cells using SPADE**

Typically, SPADE begins by performing a density-dependent down-sampling of the raw dataset followed by an unsupervised agglomerative hierarchical clustering to identify distinct sub-populations. It then builds a minimum spanning tree representation to link the clusters beginning with a randomly chosen but already connected subgraph and adding an edge to it iteratively. Finally, it performs up-sampling by assigning all cells in the initial dataset to the clusters identified (22).

Ideally, SPADE can recover cellular hierarchy corresponding to known biology from high-dimensional cytometry data-sets. However, performance is limited by a number of user-defined factors such as the desired number of clusters, outlier density, and target density following down-sampling which can affect the detection of rare cells. Furthermore, since SPADE is a non-deterministic method and the minimum-operation in the spanning-tree step is sensitive with respect to outliers, every run would result in a distinctly different tree structure.

**Figure 3:**
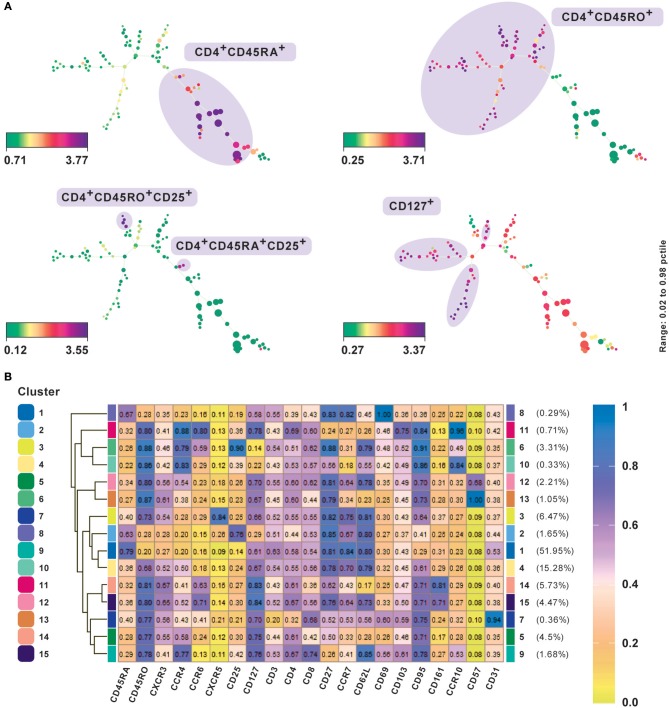
Subset classification of single cells by unsupervised clustering using SPADE and FlowSOM. **(A)** SPADE analysis with publicly available original CyTOF data from CD4^+^ human PBMC (20). Nodes in SPADE trees were overlaid with CD45RA, CD45RO, CD25, and CD127 marker expression for characterization of naïve (CD45RA^+^), memory (CD45RO^+^), as the two main branches, and regulatory T cell or Treg (CD25^hi^CD127^lo^) populations. Multiple SPADE runs result in slightly differing trees, however, the cellular subtypes identified between runs with the same dataset are similar. The tree structure, as well as connectivity via edges, also give information regarding the degree of similarity between cell populations. **(B)** FlowSOM clustering performed on the same data. Data was transformed using logicle transformation. Median intensities of 21 markers were used for analysis. FlowSOM provides a concise representation of the potential number of cell types present in the study. It can be used as a starting point for data exploration prior to biaxial gating. Meta-clustering with 15 clusters was able to identify the expected clusters. Additionally, by not down-sampling the raw data, it could potentially identify low frequency clusters as well, such as CD57^+^ and CD31^+^cells, which gives FlowSOM an advantage in being able to capture subtle differences between clusters based on their differential marker profiles.

A typical SPADE tree is comprised of nodes representing cell clusters, which are further connected through edges, which represent relationships and provide information about the underlying similarity of cell-types (22). Only the connections between nodes via edges can be used to draw conclusions about cluster similarities. The larger the distance between two connected clusters, the more dissimilar are the features of the events within those clusters. Additionally, a SPADE tree can be colored using the expression level of any preferred marker giving insights into the differential expression pattern between the events from different clusters. We analyzed the performance of SPADE using the CD4^+^ T cell CyTOF data set from PBMC (20). The data was transformed using the hyperbolic arcsine function. SPADE was applied to this dataset using all the surface markers and default parameter settings except for the number of clusters, which was set at 100 to better capture the heterogeneity of the data. To explore the underlying structure and heterogeneity in the data the SPADE trees were annotated using median expression of different markers (Figure 3A). The median marker intensities for CD45RA and CD45RO clearly indicate the presence of a naïve and memory T cell compartment in the peripheral blood. Additionally, by comparing expression of markers CD25 and CD127 one could also identify and delineate regulatory T cells from naïve and central memory T cells and effector T cells. SPADE is implemented as an R package and is also available from Cytobank and FlowJo.

A central challenge in visualizing larger datasets is to achieve and maintain performance without compromising on speed. In line with this, Van Gassen et al. (11) introduced FlowSOM, which uses self-organizing maps (Box 3). In contrast to t-SNE and SPADE analyses, several plots are not required to determine an accurate cell-type classification of clusters and their boundaries can be determined from a single heatmap of marker expression intensities (Figure 3A) or star chart map.

Box 3 **Self-organizing maps of single-cells**

Self-organizing maps (SOMs) are a classical unsupervised dimensionality reduction and clustering technique, where continuous variables (e.g., expression values or marker intensities) are used to produce discrete values. A “map” (an artificial neural network) is trained on this representation of the input space to produce a low-dimensional embedding of the same. SOMs are designed to preserve the topological information of the original input space by mapping similar high-dimensional data points to the same region in 2D space (23).

SOMs in FlowSOM are trained on the input matrix which performs the embedding of the higher dimensional space onto a rectangular grid. The resulting grid of nodes already correspond to cells, clustered using nearest neighbors, and can be visualized as star charts using mean marker expressions. The nodes of the self-organizing map are connected in a minimum-spanning tree for graphical representation, providing results comparable to SPADE visualizations. An additional meta-clustering step is performed, which includes a much larger number of nodes than clusters to give a detailed overview of the data with subtle differences. FlowSOM can be used alongside a manual gating analysis to easily compare the results in addition to examining events which are traditionally “gated out.” FlowSOM clustering does not mandate a reduction of data points by subsetting or down-sampling because it scales easily. Thus, by visualizing all cells simultaneously, annotating cell types become easier and the risk to lose novel cell populations is reduced.

We present results of FlowSOM clustering, which was applied to an original CD4^+^ PBMC CyTOF data set and expected to identify the known cell populations in the study (20). The data was transformed using logicle transformation and scaled. FlowSOM was applied using the standard parameter settings and lineage markers for clustering, which we considered could positively delineate subsets. Notably, the method was able to detect both high as well as low frequency cell populations (Figure 3B). Meta-clustering with 15 clusters was able to identify the expected clusters associated with naive (CD45RA^+^), memory (CD45RO^+^), Th1 (CXCR3^+^CCR4^−^), Th2 (CXCR3^−^CCR4^+^), Treg cells, follicular helper T cells (CXCR5^+^), and Th17 (CCR6^+^CD161^+^). By averting down-sampling, it could potentially identify low frequency clusters as well, such as CD57^+^ and CD31^+^ cells (clusters 12 and 13 and cluster 7, respectively), giving FlowSOM an advantage in being able to capture subtle differences between clusters based on their differential marker profiles.

## Trajectory Inference of Differentiating Cells and Graph Abstraction

Cellular differentiation is a non-linear and continuous phenomenon. Additional information can be gained from aligning asynchronously differentiating cells according to their inherent developmental state. Their temporal order can be computed from expression profiles and measured using a random-walk-based distance metric known as diffusion pseudo time (DPT) (12) (Box 4). DPT identifies developmental progression, branching points as well as differential expression of key decision-making cell biomarkers on the single-cell level. DPT has been used to analyze an InDrop single-cell RNAseq data from Klein et al. (25), where it revealed differentiation and transcription factor dynamics of mouse embryonic stem cells after leukemia inhibitory factor withdrawal and identified major clusters with different biological functions. Notably, the analysis identified one cluster enriched for pluripotency factors that were active during early pseudo-time (12).

Box 4 **Inferring cellular trajectories using diffusion pseudotime**

The first method to quantitatively estimate the progress of a cell along some biological or developmental pathway was Monocle and termed it pseudotime (24). To obtain a cell's pseudotime, the DPT algorithm first computes a transition matrix from single-cell expression data by convolving Gaussian kernels centered at nearby cells, i.e., “overlaying” the two Gaussian functions representing pairs of cells, effectively constructing a weighted nearest-neighbor graph of the data (9). Next, it determines the probabilities for each cell to transition to each other cell in the data set using random walks of any length on this graph. These transition probabilities correspond to edge weights. The random walks can be considered as a proxy for the cells' probabilities of differentiating toward another cell. The probabilities for each cell are stored in a vector, and the DPT between two cells is calculated as the Euclidean distance between their two vectors. The developmental progression of each cell in the data set is then measured by computing its DPT with respect to a specified root cell (12).

This method reveals new biology from a variety of experimental settings through robust computation of pseudo-time and scalability. In comparison to previous algorithms for pseudotemporal ordering, such as Wanderlust/Wishbone (23, 26) and Monocle (24), DPT's random-walk-based formulation has been shown to perform significantly better in ordering cells according to pseudotime. Unlike DPT, Monocle utilizes an only partially robust minimum spanning tree approach and is unable to scale to high cell numbers. Wishbone, on the other hand, computes pseudotime distance based on shortest paths on graphs, which leads to a complicated and iterative computation to account for branches (27). Since then many more algorithms for trajectory inference have been proposed, especially for scRNAseq. Saelens et al. (28) perform an extensive and comprehensive assessment of 29 published trajectory inference methods on both simulated as well as real datasets and provide a set of guidelines for users.

We evaluated the performance of diffusion maps and DPT on the CD4^+^ T cell CyTOF dataset (20) after following a density dependent down-sampling and using logicle transformation (Figures 4A,B). The root cell was chosen as the cell having the minimum expression for CD45RO, based on the knowledge that naïve T cells express CD45RA and that upon antigen exposure they differentiate into central and effector memory T cells gaining expression of CD45RO and losing expression of CD45RA (Figure 4C). The ensemble of diffusion plots clearly revealed the major T cell subsets, e.g., transition from naïve to memory T cells with Treg cells and T helper subsets originating toward the end of differentiation. The heat-map orders cells by DPT and depicts protein marker dynamics with cells transitioning from CD45RA^+^ to CD45RO^+^ (Figure 4D).

**Figure 4:**
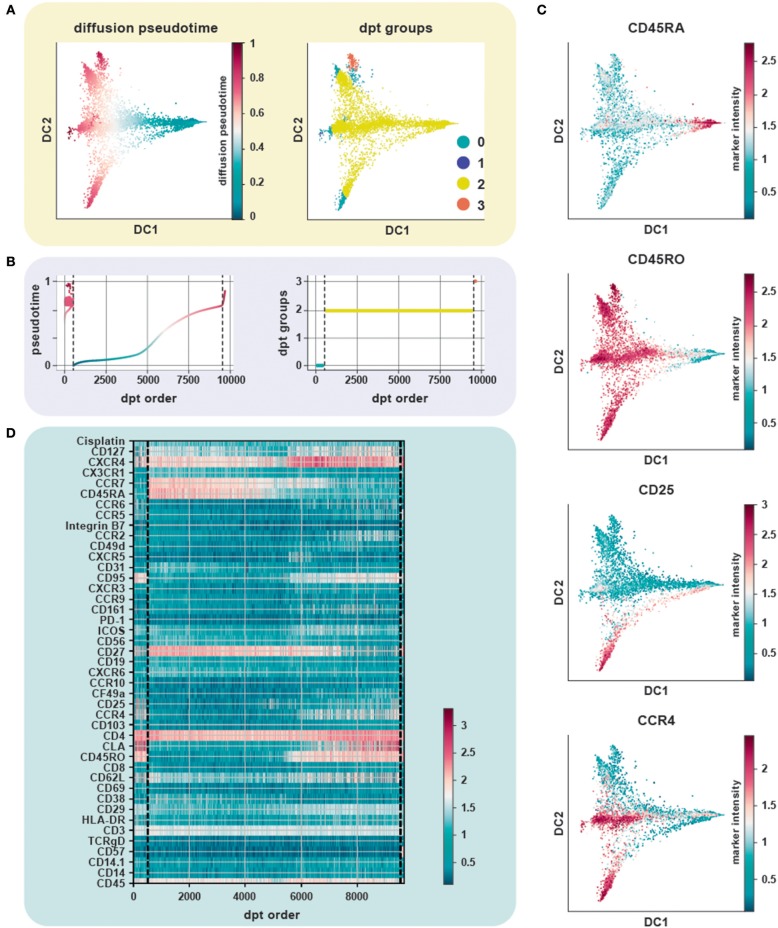
Trajectory inference from static snapshot cytometry data. **(A)** Application of DPT to CD4^+^ PBMC using an original CyTOF dataset (20). Clustering based on the tip cells identifies three major DPT groups. The root cell, with a high expression for CD45RA (naïve compartment), has the lowest pseudotime. **(B)** The three DPT groups and their corresponding changes in pseudotime. **(C)** Heat-map of marker expression with cells ordered by DPT and depicts protein marker dynamics with cells transitioning from CD45RA^+^ to CD45RO^+^ as seen in DPT group 2. **(D)** Diffusion maps of CyTOF data colored by selected lineage marker intensity profiles, which identify major immune subsets aligned along internal developmental states of the data. The ensemble of diffusion plots clearly reveals the major T cell subsets, e.g., cells differentiating from a naïve to a memory state with Treg cells, T helper subsets such as Th1 and Th2 cells populating the ends of the trajectory.

Many unsupervised single-cell data analysis algorithms are based on clustering approaches which label groups of cells into discrete clusters with biologically distinct phenotypic and functional characteristics. On the other hand, trajectory inference algorithms assume that data lie on a connected manifold and project cells on a so-called pseudotime by computing paths between them using some distance metric along this manifold. Partition-based graph abstraction (PAGA) combines analysis strategies of both clustering as well as trajectory modeling to compute an abstracted graph representing the overall topology of a possibly disconnected manifold of cells (13). It first computes a neighborhood graph of single cells whose partitions represent groups of similar cells. From this, it generates a simple abstracted graph whose nodes correspond to these partitions and edges represent a confidence measure for the connectivity between partitions. The method utilizes a random-walk-based distance measure to generate a topology preserving map of the underlying differentiation manifold from single-cell measurements and is shown to be computationally efficient (13).

We applied PAGA on the CD4^+^ T cell PBMC CyTOF dataset using default settings and a resolution of 0.6 (Figure 5A) (20). The abstracted graph was able to reconstruct the major T cell subsets arising from CD4^+^ T helper cell differentiation. Cluster 1, 2, 3, and 4 constituted the memory compartment while cluster 0 and 5 represent naïve subsets. Cluster phenotypes could be identified from the marker expression distribution (Figure 5B). In datasets with inherent continuous manifolds, PAGA constructs a tree-like lineage graph with disconnected clusters. Together it explains the global topology of the data and also reconstructs differentiation processes.

**Figure 5:**
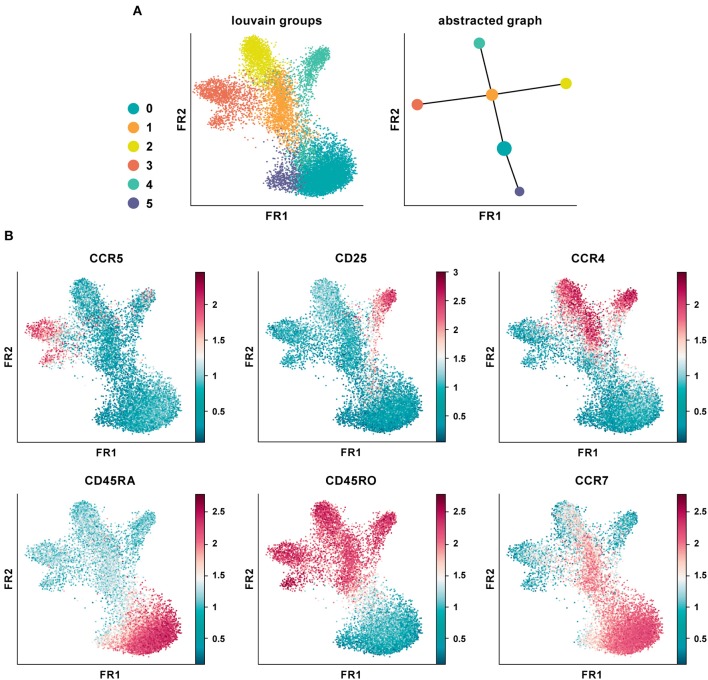
Partition-based graph abstraction of CyTOF data. **(A)** Shown is a data analysis of an original CyTOF data-set depicting CD4^+^ T cells derived from PBMC (20). Louvain groups in a single-cell graph visualized using the Fruchtman-Reingold (FR) algorithm, which conserves continuous structure in data better than t-SNE. Abstracted graph visualized using a simple tree-based graph drawing layout. **(B)** Identification of cluster phenotypes from marker expression distribution. In data-sets with inherent continuous manifolds, PAGA constructs a tree-like lineage graph with disconnected clusters. Together, it explains the global topology of the data as well as reconstructs differentiation processes.

## Considerations for the Choice of Appropriate Algorithms

The choice of the most informative type of analysis is dictated by the respective question to be addressed. Among clustering tools, FlowSOM currently achieves the top benchmarking results for typical CyTOF analyses and falls behind only slightly in the detection of rare cell populations (29). While in general results from FlowSOM are robust in terms of reproducibility some caution needs to be taken of rare “outlier runs.” The heatmap organization allows for a compact overview of the entire marker set facilitating cluster annotation, and of the distance of clusters based on equal-weighted marker contributions.

Although computation-light, PCA as a linear dimensionality reduction approach is typically less powerful than non-linear dimensionality reduction methods when applied to CyTOF data. In the latter category, UMAP has advantages over SNE in preserving both local and global distances among cells (14). Strikingly, this does not come at the cost of greater computational load but is competitive also to the latest benchmarked versions of SNE-based algorithms. UMAP can recapitulate known developmental trajectories, e.g., in haematopoeisis, yet more specialized approaches may be preferred to infer (pseudo)temporal ordering in less well-defined differentiation processes.

This additional layer of information can be extracted from single-cell data through trajectory inference, which adds dynamics to static snapshot data. The diffusion maps allow for the detection of developmental trajectories without neglecting rare populations and, through placing cells in a pseudotemporal order, they allow for inference on differentiation stages. Even though this method is more robust than t-SNE in coping with noise, it was applied only for visualization purposes. To cover that gap, DPT was developed with the capacity of measuring the transitions between developmental stages to depict fate decisions on a diffusion map. In addition, it can be scaled to higher cell numbers, differently from Monocle, without large computational requirements as observed for Wanderlust and Wishbone. PAGA is also very computationally efficient and has the advantage of dealing well with cells disconnected in the pseudotime, thus reflecting the absence of some intermediary developmental stages in the sample.

## Concluding Remarks and Future Perspectives

Single-cell technologies have become a ubiquitous tool as researchers realize their untapped potential to uncover cellular heterogeneity and functionality at a greater resolution than with bulk analysis. The past few years have seen a significant change in the ways cytometry datasets are being analyzed and a wealth of novel computational tools is now available to mine complex and high-dimensional data in an unbiased automated manner. Depending on the biological question, one of several computational methods can be incorporated to potentially substantiate the findings of manual gating as well as deepen our understanding of how the immune system functions in health and disease.

Many integrated data analysis frameworks now exist to facilitate a comprehensive interrogation of high-dimensional single-cell data. Most of these have been originally developed for scRNA-seq data. However, they can be extended for the purpose of cytometry as well. Cytofkit, an integrated analysis pipeline, is specially designed for mass cytometry data and is available as a Bioconductor package (30). It also provides a graphical user interface as well as a Shiny application for interactive and effortless usage and visualization of results. Seurat, also available as a Bioconductor package, contains implementations of commonly applied analytical techniques for exploring single-cell expression data (30). Scanpy, a Python frame-work, provides computationally efficient and state-of-the-art methods to address the statistical challenges associated with scRNA-seq data (31). All of these packages incorporate both novel as well as established methods to perform data pre-processing, feature selection, linear and non-linear dimensionality reduction, standard unsupervised clustering algorithms for automatic detection of cell subsets and differential testing. Scanpy additionally integrates novel in-house algorithms and performs trajectory inference. One of the most productive research areas in future should be toward developing and maintaining such integrated analysis pipelines for cytometry data as well as bridging the gap with other OMICS data analysis for a more comprehensive interpretation of study models.

There are many more established algorithms for single-cell analysis mostly developed for scRNA-seq that also allow investigation of cytometry datasets, several of which have been reviewed earlier (5, 32, 33); for a large-scale overview please consider this list (34). However, we find that the different methods vary significantly in terms of scalability, speed and computational skill required to interpret results. We discuss and demonstrate the feasibility and power of several current computational tools to translate complex static snapshot data obtained from high-dimensional single-cell datasets into dynamic ontological and regulatory networks of the immune system. A potential avenue for further development would be to incorporate machine learning methods to infer developmental trajectories directly from cytometry data, which currently describes much less features to model the underlying manifold and is, thus, a limitation. Ultimately, it depends on the experience and requirement of the investigator to make an informed decision about the choice of the data exploratory method.

## Author Contributions

SP drafted the manuscript and analyzed the data. CH and GdA drafted the manuscript. FT critically reviewed the data analysis and manuscript. CZ provided the concept of the manuscript and directed the data analysis, designed the figures, and critically reviewed the manuscript.

### Conflict of Interest Statement

The authors declare that the research was conducted in the absence of any commercial or financial relationships that could be construed as a potential conflict of interest.

## References

[1] Mahnke YD Roederer M Optimizing a multicolor immunophenotyping assay. Clin Lab Med. (2007) 27:469–85. 10.1016/j.cll.2007.05.002 17658403 PMC2034273

[2] Bandura DR Baranov VI Ornatsky OI Antonov A Kinach R Lou X Mass cytometry: a novel technique for real-time single cell multi-target immunoassay based on inductively coupled plasma time of flight mass spectrometry. Analyt Chem. (2009) 81:6813–22. 10.1021/ac901049w 19601617

[3] Spitzer MH Nolan GP Mass cytometry: single cells, many features. Cell. (2016) 165:780–91. 10.1016/j.cell.2016.04.019 27153492 PMC4860251

[4] Roederer M Spectral compensation for flow cytometry: visualization artifacts, limitations, and caveats. Cytometry. (2001) 45:194–205. 10.1002/1097-0320(20011101)45:3<194::AID-CYTO1163>3.0.CO;2-C 11746088

[5] Mair F Hartmann FJ Mrdjen D Tosevski V Krieg C Becher B The end of gating? An introduction to automated analysis of high dimensional cytometry data. Eur J Immunol. (2016) 46:34–43. 10.1002/eji.201545774 26548301

[6] Aghaeepour N Finak G. The FlowCAP consortium, The DREAM Consortium Hoos H Mosmann TR Critical assessment of automated flow cytometry data analysis techniques. Nat Methods. (2013) 10:228–38. 10.1038/nmeth.2365 23396282 PMC3906045

[7] Liu S Trapnell C Single-cell transcriptome sequencing: recent advances and remaining challenges. F1000Research. (2016) 5:F1000 Faculty Rev-182 10.12688/f1000research.7223.1 PMC4758375 26949524

[8] Amir EAD Davis KL Tadmor MD Simonds EF Levine JH Bendall SC ViSNE enables visualization of high dimensional single-cell data and reveals phenotypic heterogeneity of leukemia. Nat Biotechnol. (2013) 31:545–52. 10.1038/nbt.2594 23685480 PMC4076922

[9] Haghverdi L Buettner F Theis FJ Diffusion maps for high-dimensional single-cell analysis of differentiation data. Bioinformatics. (2015) 31:2989–98. 10.1093/bioinformatics/btv325 26002886

[10] Qiu P Simonds EF Bendall SC Gibbs KD Bruggner RV Linderman MD Extracting a cellular hierarchy from high-dimensional cytometry data with SPAD. Nat Biotechnol. (2011) 29:886–91. 10.1038/nbt.1991 21964415 PMC3196363

[11] Van Gassen S Callebaut B Van Helden MJ Lambrecht BN Demeester P Dhaene T Using self-organizing maps for visualization and interpretation of cytometry data. Cytometr A. (2015) 87:636–45. 10.1002/cyto.a.22625 25573116

[12] Haghverdi L Büttner M Wolf FA Buettner F Theis FJ Diffusion pseudotime robustly reconstructs lineage branching. Nat Methods. (2016) 13:845–8. 10.1038/nmeth.3971 27571553

[13] Wolf FA Hamey F Plass M Solana J Dahlin JS Gottgens B Graph abstraction reconciles clustering with trajectory inference through a topology preserving map of single cells. BioRxiv. [Preprint] (2017). 10.1101/208819 PMC6425583 30890159

[14] Becht E McInnes L Healy J Dutertre CA Kwok IWH Ng LG Dimensionality reduction for visualizing single-cell data using UMAP. Nat Biotechnol. (2018) 37:38–44. 10.1038/nbt.4314 30531897

[15] McInnes L Healy J UMAP: uniform manifold approximation and projection for dimension reduction. arXiv:1802.03426v2 (2018).

[16] Van Unen V Höllt T Pezzotti N Li N Reinders JTM Eisemann E Visual analysis of mass cytometry data by hierarchical stochastic neighbour embedding reveals rare cell types. Nat Comm. (2017) 8:1740 10.1038/s41467-017-01689-9 PMC5700955 29170529

[17] Höllt T Pezzotti N van Unen V Koning F Eisemann E Lelieveldt B Cytosplore: interactive immune cell phenotyping for large single-cell datasets. Comp Graph Forum. (2016) 35:171–80. 10.1111/cgf.12893

[18] van der Maaten L Barnes-Hut-SNE. arXiv:1301.3342v2 (2013).

[19] Wattenberg M Viégas F Johnson I How to use t-SNE effectively. Distill. (2016). 10.23915/distill.00002

[20] Wong MT Ong EHD Lim SHF Teng WWK McGovern N Narayanan S A high-dimensional atlas of human T cell diversity reveals tissue-specific trafficking and cytokine signatures. Immunity. (2016) 45:442–56. 10.1016/j.immuni.2016.07.007 27521270

[21] Coifman RR Lafon S Diffusion maps. Appl Comp Harmon Anal. (2006) 21:5–30. 10.1016/j.acha.2006.04.006

[22] Anchang B Hart DPT Bendall SC Qiu P Bjornson Z Linderman M Visualization and cellular hierarchy inference of single-cell data using SPADE. Nat Protocols. (2016) 11:1264–79. 10.1038/nprot.2016.066 27310265

[23] Kohonen TJBC Self-organized formation of topologically correct feature maps. Biol Cybernet. (1982) 43:59–69. 10.1007/BF00337288

[24] Trapnell C Cacchiarelli D Grimsby J Pokharel P Li S Morse M The dynamics and regulators of cell fate decisions are revealed by pseudo-temporal ordering of single cells. Nat Biotechnol. (2014) 4:381–6. 10.1038/nbt.2859 PMC4122333 24658644

[25] Klein AM Mazutis L Akartuna I Tallapragada N Veres A Li V Droplet barcoding for single-cell transcriptomics applied to embryonic stem cells. Cell. (2015) 161:1187–201. 10.1016/j.cell.2015.04.044 26000487 PMC4441768

[26] Bendall SC Davis KL Amir ADE Tadmor MD Simonds EF Chen TJ Single-cell trajectory detection uncovers progression and regulatory coordination in human B cell development. Cell. (2014) 157:714–25. 10.1016/j.cell.2014.04.005 24766814 PMC4045247

[27] Setty M Tadmor MD Reich-Zeliger S Angel O Salame TM Kathail P Wishbone identifies bifurcating developmental trajectories from single-cell data. Nat Biotechnol. (2016) 34:637–45. 10.1038/nbt.3569 27136076 PMC4900897

[28] Saelens W Cannoodt R Todorov H Saeys Y A comparison of single-cell trajectory inference methods: towards more accurate and robust tools. BioRxiv. [Preprint] (2018). 10.1038/s41587-019-0071-9 30936559

[29] Weber LM Robinson MD Comparison of clustering methods for high-dimensional single-cell flow and mass cytometry data. Cytometry A. (2016) 89:1084–96. 10.1002/cyto.a.23030 27992111

[30] Butler A Hoffman P Smibert P Papalexi E Satija R Integrating single-cell transcriptomic data across different conditions, technologies, and species. Nat Biotechnol. (2018) 36:411–20. 10.1038/nbt.4096 29608179 PMC6700744

[31] Wolf FA Angerer P Theis FJ SCANPY: large-scale single-cell gene expression data analysis. Genome Biol. (2018) 19:15 10.1186/s13059-017-1382-0 29409532 PMC5802054

[32] Kimball AK Oko LM Bullock BL Nemenoff RA van Dyk LF Clambey ET A Beginner's guide to analyzing and visualizing mass cytometry data. J Immunol. (2018) 200:3–22. 10.4049/jimmunol.1701494 29255085 PMC5765874

[33] Newell EW Cheng Y Mass cytometry: blessed with the curse of dimensionality. Nat Immunol. (2016) 17:890–5. 10.1038/ni.3485 27434000

[34] Rostom R Svensson V Teichmann SA Kar G Computational approaches for interpreting scRNA-seq data. FEBS Lett. (2017) 591:2213–25. 10.1002/1873-3468.12684 28524227 PMC5575496

